# Family screening for genetic cardiomyopathies in ECG-positive phenotype-negative adolescents: a joint clinical and genetic approach

**DOI:** 10.1007/s00431-026-07524-y

**Published:** 2026-10-19

**Authors:** Mark Abela, Jessica Debattista, Jeanesse Scerri, Tiziana Felice, Victor Grech, Christian Scerri

**Affiliations:** 1 Department of Cardiology, Mater Dei Hospital, Msida, MSD2090 Malta; 2 University of Malta, Msida, Malta; 3 Cardiovascular and Genomics Research Institute at St George’s, University of London, London, UK; 4 Molecular Genetics, University of Malta, Msida, Malta; 5 Department of Paediatrics, Mater Dei Hospital, Msida, Malta

**Keywords:** Family screening, ECG-positive phenotype-negative, Pre-clinical, ECG, Genetics, Cardiomyopathy, Adolescent

## Abstract

Adolescents may present with abnormal ECG findings despite having no structural or electrical evidence of cardiomyopathy. The value of extending family screening to this subgroup remains uncertain. To evaluate the impact of family screening in ECG-positive phenotype-negative adolescents, and determine whether a joint clinical-genetic approach improves the diagnostic yield. In this prospective cohort study, adolescents with an abnormal ECG identified through a nationwide screening program underwent comprehensive clinical/genetic evaluation. Phenotype-negative individuals were included. Relatives underwent clinical screening, with cascade genetic testing offered when probands carried a likely/definite pathogenic (LP/P) cardiomyopathy variant. Abnormal screening outcomes included relatives with (i) de novo cardiomyopathy diagnosis, (ii) early cardiomyopathic traits, or (iii) gene-positive/phenotype-negative (G + P −) status. Sixteen adolescents (0.6%) met inclusion criteria, and 3 (18.8%) were G + (MYBPC3, MYH7, ACTC1). One in five relatives (*n* = 13/71, 18.3%; 95% CI 11.0–28.9%) were referred, most having an abnormal ECG (12.7%). Nine relatives (12.7%; 95% CI 4.9–20.4%) exhibited early cardiomyopathy traits at baseline. One (1.4%) relative was G + /P − . Cardiomyopathy was diagnosed in 3 (4.2%, 95% CI 0.9–11.8%) relatives at baseline (*n* = 2 HCM, *n* = 1 DCM). One relative with early cardiomyopathy traits underwent phenoconversion (NDLVC) during follow-up at 77 months, yielding an overall diagnostic rate of 5.6% (95% CI 2.0–13.8%) in 4 unrelated families. A joint clinical-genetic approach enabled personalized surveillance in 6 relatives (8.5%), including 2 (2.8%) diagnoses that would otherwise have been missed with conventional clinical screening.

*Conclusion*: One in five relatives of ECG‑positive phenotype‑negative adolescents received a cardiomyopathy diagnosis or had early cardiomyopathy traits. A joint clinical-genetic approach in this subgroup may aid early cardiomyopathy detection in selected families, though findings remain exploratory and require validation in larger cohorts.

**Table:** 

**What is Known – What is New:** *•Resting ECG can identify individuals harboring conditions linked to sudden cardiac death* *•The penetrance of cardiomyopathy is age dependent, which explains why screened adolescents may exhibit abnormal ECG patterns in the absence of a cardiomyopathy diagnosis* *•Our data indicate that extending screening to relatives of ECG-positive, phenotype-negative adolescents is justified, with one in five relatives receiving a new cardiomyopathy diagnosis (5.6%) or exhibiting early cardiomyopathy traits (11.3%)* *•A joint clinical/genetic evaluation in this context may help personalize screening strategies*

**What is Known – What is New:**

*•Resting ECG can identify individuals harboring conditions linked to sudden cardiac death*

*•The penetrance of cardiomyopathy is age dependent, which explains why screened adolescents may exhibit abnormal ECG patterns in the absence of a cardiomyopathy diagnosis*

*•Our data indicate that extending screening to relatives of ECG-positive, phenotype-negative adolescents is justified, with one in five relatives receiving a new cardiomyopathy diagnosis (5.6%) or exhibiting early cardiomyopathy traits (11.3%)*

*•A joint clinical/genetic evaluation in this context may help personalize screening strategies*

## Introduction

Sudden cardiac death (SCD) in young individuals is a tragic unexpected event that provokes profound emotional stress for the victim’s loved ones. Up to 10% of all sudden deaths in the UK occur in adolescent individuals, with sudden arrhythmic death syndrome and myocardial disorders being the most common conditions diagnosed at autopsy [1]. There are no prewarning symptoms or a relevant family history in up to 80% of cases [2, 3]. Most deaths are preventable, with the pathologies linked to SCD often identified during cardiac screening with a resting 12-lead electrocardiogram (ECG) [4]. Several groups have reported an increased risk of SCD during elite sports [5–8]. This has led to most sporting bodies mandating ECG-based cardiac screening in their athletes [9].

The benefits of such an approach in athletes have paved the way to extending cardiac screening programs to non-athletes [10, 11]. Screening younger individuals however has caveats. Pre-clinical phenotypes in this age bracket are frequently encountered, and phenotypic markers of disease are often subtle and non-diagnostic [12]. False negatives using this approach are also inevitable, as disease penetrance, notably in cardiomyopathies, is age dependent. These screening caveats were experienced first-hand in a nationwide cardiac screening program for adolescents, with most individuals with an abnormal ECG having no clinical diagnosis [11]. Repeated screening in young athletic individuals has however been shown to improve the diagnostic yield [13], highlighting the importance of surveillance in grey zone cases.

Cardiomyopathy is a leading cause of SCD, hence why family screening is one of the key pillars in the management of these families. Guidelines currently advocate pursuing family screening and genetic testing in individuals fulfilling criteria for a hereditary disorder [12, 14]. This extended familial evaluation better informs physician of disease expressivity in families, and also helps diagnose other affected individuals [15–17]. Phenotype and diagnosis are often used interchangeably in consensus documents. Phenotype is actually from the Greek word “*phainein*” meaning “appear” and “*typos*” meaning “type,” referring to morphological traits of a disease. Criteria for diagnosis are often very stringent, following a binary metric. Disease expression in cardiomyopathy is however highly heterogenous, with ECG anomalies, and morphofunctional or electrical abnormalities frequently reported in cardiomyopathy gene carriers, even though they may not be fulfilling diagnostic criteria for cardiomyopathy [18–24]. Data emerging from genetic repositories has reinvigorated interest in precision medicine, with some groups now advocating for a genotype-first approach [18, 25]. Some have floated the idea that a combined clinical and genetic evaluation may be reasonable in individuals harboring non-diagnostic clinical phenotypes [26]. A gene-positive (G +) status is also an important predictor for early onset and more aggressive disease [17, 25, 27, 28]. Data from the UK biobank has also shown that 1 in 6 adults with putative pathogenic variants in dilated cardiomyopathy (DCM) genes exhibited early DCM features on ECG or cardiac magnetic resonance (CMR), abnormalities which in isolation may not necessarily be diagnostic [29]. Most cardiomyopathies present in the 3rd or 4th decades of life, which is why the penetrance of cardiomyopathy for most genetic variants is low in children and adolescents [30]. There is also minimal and infrequent progression to an overt phenotype in the short term [31–36]. The penetrance is however higher in families with a genetic variant compared to gene elusive families [32, 35]. Naturally, not all G + _ individuals harboring a LP/P variant end up with a diagnosis [19, 37–39], which is why one should exercise caution when counseling gene carriers. The ramifications of a positive genetic result can undoubtedly led to significant emotional disturbances, alongside potential future impacts on career prospects, financial stability, exercise routines, and family planning [18, 40].

Family screening has traditionally not been adopted in individuals harboring pre-clinical phenotypes. Early detection and management of asymptomatic young individuals which may potentially be harboring Mendelian cardiovascular disease through screening may on the other hand improve outcomes. This may help determine the most appropriate surveillance and treatment strategy, while also better informs clinicians and families on the potential risks [41].

Despite increasing recognition of early phenotypic markers and the potential value of genetic testing, there remains limited evidence to guide family screening in adolescents who present with an abnormal ECG but do not meet diagnostic criteria. To address this knowledge gap, the main objective of this study was to explore the diagnostic yield of a family screening program in ECG-positive phenotype-negative probands, potentially suggestive of pre-clinical cardiomyopathy. A secondary aim was to determine whether incorporating genetic testing in these probands led to an improved overall diagnostic rate among their family members.

## Methodology

This was a single-center, prospective cohort study [11]. Adolescents were systematically invited to undergo cardiac screening as part of a nationwide cardiac screening program (BEAT-IT) in 2017–2018) [11]. The screening protocol consisted of a health questionnaire and resting 12-lead ECG. Individuals who gave consent (including parental consent) were enroled into the screening program. The health questionnaire and ECG were reviewed by a physician on site during school hours. The international criteria for ECG interpretation were used for reporting ECG [42]. All ECG were reviewed by two independent cardiologists. Adolescents with an abnormal health questionnaire and/or an abnormal ECG were referred for comprehensive clinical evaluation, as previously published [11]. For the purpose of this study, adolescents with an abnormal ECG suggestive of cardiomyopathy, in the absence of a clinical diagnosis (pre-clinical phenotype), were included. The principal investigator informed these adolescents of the possibility that their relatives could undergo family screening. Relatives who wished to be evaluated were asked to contact the research team directly. Those with an abnormal screen underwent a multi-modality assessment (next section).

**Fig. 1:**
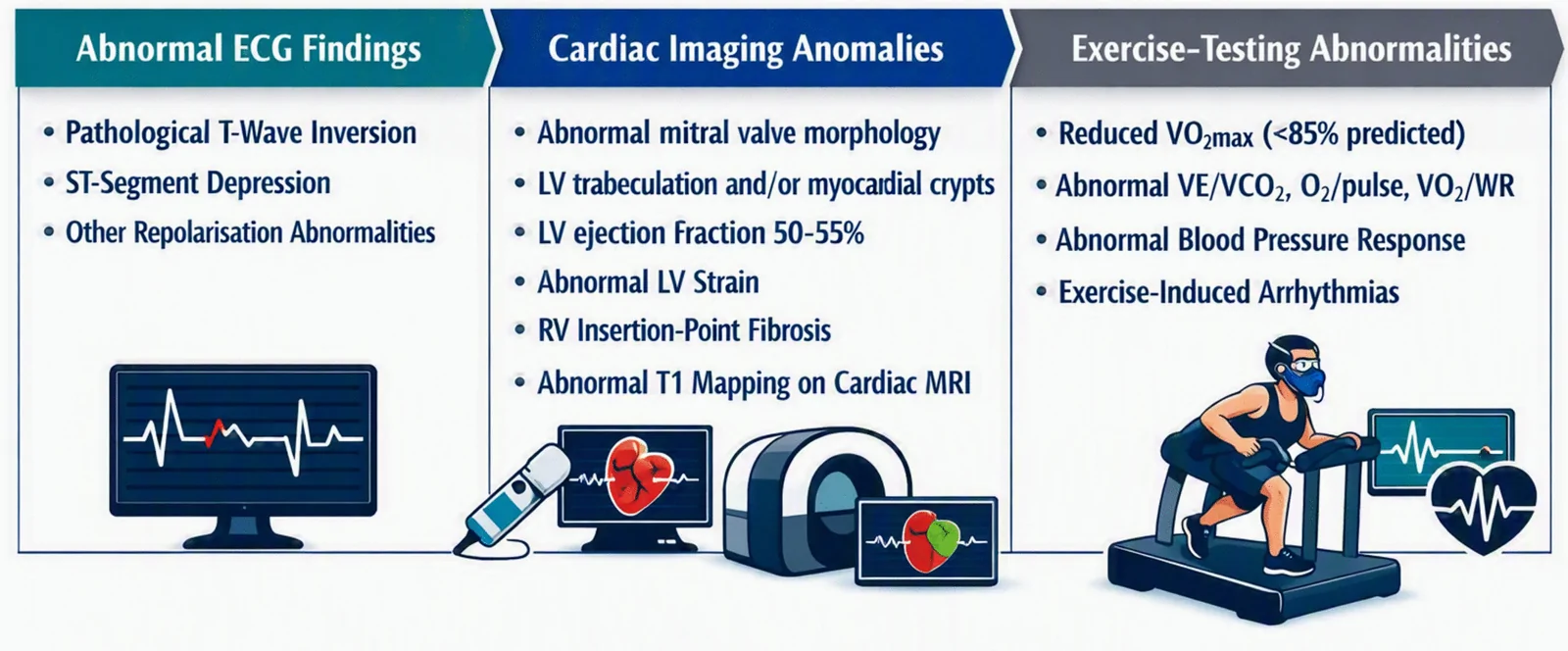
Defining early cardiomyopathy traits

An abnormal evaluation in screened relatives included those with (a) a definite cardiomyopathy diagnosis fulfilling guideline criteria [12, 14, 43–45]; (b) early cardiomyopathy traits (Fig. 1) requiring surveillance (pathological ECG patterns, morphofunctional anomalies on cardiac imaging, and/or abnormalities during cardiopulmonary exercise testing [CPET]); and (c) and gene-positive phenotype-negative (G + /P −) harboring a LP/P cardiomyopathy variant in the absence of early cardiomyopathy traits or diagnosis. All patients were offered surveillance and counseling by expert physicians.

### Probands – screening protocol

Adolescents with a resting 12-lead ECG suggestive of cardiomyopathy, without a clinical diagnosis, were included for this study. An abnormal ECG suggestive of cardiomyopathy included individuals with two or more borderline ECG abnormalities (axis deviation, atrial enlargement) and/or pathological ECG patterns (anterior T wave inversion (≥ 16 years), inferior/lateral TWI, pathological Q waves [excluding lead III, aVR], ST segment depression, left bundle branch block, right bundle branch block [QRS duration ≥ 140 ms], ≥ 2 premature ventricular/atrial beats [PVBs/PACs]) [42]. The multi-modality assessment performed to rule out cardiomyopathy included a repeat resting 12-lead ECG, an echocardiogram, exercise tolerance test, 24-h ambulatory ECG monitoring, and CMR imaging. Part of the work-up also included a cardiac gene panel. Expert genetic counseling led by a multidisciplinary team (consisting of a cardiologist with expertise in inherited cardiac conditions, geneticists, and genetic counselors) was carried out. These subjects were counseled on the possible advantages and negative ramifications of genetic testing. Participants were informed that surveillance remained imperative, irrespective of the outcome of genetic testing. Individuals were however informed that the presence of a LP/P cardiomyopathy variant may alter the surveillance strategy during follow-up (frequency and advanced imaging modalities). The presence of a LP/P variant would also play a role in cascade testing of family members. Parental and patient consent was mandatory before testing. A 218 cardiac gene panel (Health in Code S.L., A Coruna, Spain) (supplementary text) was performed on all included probands. Variants were classified as variants of unknown significance (VUS), LP, or P according to the ACMG criteria [46], using *Franklin Genoox* to compute criteria supporting pathogenicity.

### Relatives—screening protocol

First-degree relatives of probands with an abnormal ECG suggestive of cardiomyopathy, without a clinical diagnosis, were consequently informed of the possibility of undergoing screening (recruited between 2018 and 2020). This consisted of a clinical assessment (history and examination), a resting 12-lead ECG and an echocardiogram. ECG were reported using the same criteria for probands [42]. Abnormal ECG findings suggestive of early cardiomyopathy were defined as previously described for probands. Second-line investigations were also organized for relatives with an abnormal screen. Internationally recognized diagnostic criteria were applied for those labeled with a cardiac disorder [12, 14, 45]. Following clinical testing, first-degree relatives of probands with a LP/P cardiomyopathy variant were counseled on the implications of cascade genetic testing. This approach was also extended to non-immediate family members if first-degree (or next generation) relatives were given a clinical diagnosis, harbored early cardiomyopathy traits, and/or tested positive for the familial cardiomyopathy (LP/P) variant. Probands and their relatives with an abnormal evaluation were followed up yearly in a dedicated inherited cardiac conditions clinic. Adolescent relatives were defined as individuals ≤ 18 years at the first evaluation. Relatives and probands with a LP/P genetic variant (in the absence of a diagnosis) had a repeat CMR at 5 years from baseline.

### Data collection

Medical records were reviewed for demographics, family history, baseline clinical characteristics, cardiac symptoms, diagnostic tests, and genetic evaluation. Data on those given a diagnosis or advised to undergo surveillance was recorded. Information on relatives given lifestyle advice and early medical interventions (medical therapy and cardiac interventions) was also collected. Follow-up data about evolving phenotypes and clinical outcomes were also recorded using electronic health records. A major adverse cardiac event (MACE) was defined as (i) cardiac-related hospitalization related to a genetic cardiomyopathy (diagnosed at baseline or during follow-up); (ii) cardiac implantable electronic device implantation; and/or (iii) cardiac-related death.

### Ethical approval

The study was approved by the University of Malta Faculty of Medicine Research Ethics Committee (Approval Number 34/2017) and conforms to the principles stated in the Declaration of Helsinki. Written informed consent for screening was obtained from all relatives.

### Statistical analysis

Statistical computations were carried out with SPSS V.23 (IBM, Armonk, NY, USA). Data is presented as the mean ± SD and data ranges. Categorical variables are reported as frequencies and percentages.

## Results

### Proband evaluation

A total of 2708 adolescents (mean age 15 years, 50.4% male) underwent cardiac screening. Sixteen (0.6%) were ECG-positive phenotype-negative individuals. Their ECG had changes suggestive of cardiomyopathy. No one was given a diagnosis or exhibited early cardiomyopathy traits (morphofunctional or electrical) on second line investigations (Table 1) [11]. One had a family history of SCD in a second-degree relative.

**Table 1:** Genotype-ECG correlation in probands and baseline/follow-up outcome of family screening (*LP*, likely pathogenic; *P*, pathogenic; *TWI*, T wave inversion; *VUS*, variant of unknown significance)

Family	Proband ECG	Gene	Mutation	Pathogenicity	ACMG supporting criteria	Novel	Screened relatives		
							Screened (*n*, %)	Diagnosis (*n*, %)	Surveillance (*n*, %)
1	Inferolateral TWI	ACTC1	NM_005159.4:c.469 T > C	LP	PM2, PP1, PP2, PP3	✓	11	1 (9.1)	5 (45.5)
2	Inferior TWI	FHOD3	NM_001281740.2:c.1783C > T	VUS	PM2, BS2, BP4, BP6	✗	5	0 (0.0)	0 (0.0)
3	Inferior TWI	MYH7	NM_000257.3:c.3145G > A	LP	PM2, PM1, PP2, PP3	✓	7	1 (14.3)	0 (0.0)
4	Anterior/Inferior TWI	MYBPC3	NM_000256.3:c.1591G > C	P	PS4, PP1, PS3, PS1, PM5, PM2, PP3, PP5	✗	5	1 (20.0)	0 (0.0)
5	ST depression	DSP RYR2	NM_004415.3:c.7924G > A NM_001035.2:c.10189C > T	VUS VUS	PM2, BP6 PM2, PP2, BP6	✗ ✗	5	0 (0.0)	0 (0.0)
6	Inferolateral TWI	TRIM63 DSG2	NM_032588.3:c.224G > A NM_001943.4:c.1397C > T	VUS VUS	PS4, PP3, PM2, PP5 PM2, BP4	✗ ✗	8	0 (0.0)	1 (0.0)
7	Anterolateral TWI	MYH7	NM_000257.3:c.5500G > A	VUS	PM1, PP2, PM2, BP6	✗	2	1 (50.0)	0 (0.0)
8	Inferolateral TWI	DSP ANKRD1	NM_004415.3:c.7039A > G NM_014391.2:c.461G > A	VUS VUS	PM2, PP3, BP6 PM2	✗ ✗	4	0 (0.0)	1 (25.0)
9	Anterior TWI	FLNC	NM_001458.4:c.2092G > A	VUS	PM2, PP3	✗	3	0 (0.0)	1 (33.3)
10	Inferior TWI	Negative					3	0 (0.0)	0 (0.0)
11	Inferolateral TWI	Negative					3	0 (0.0)	0 (0.0)
12	Anterior TWI	Negative					2	0 (0.0)	0 (0.0)
13	Anterior TWI	Negative					3	0 (0.0)	0 (0.0)
14	Anterior TWI	Negative					4	0 (0.0)	1 (25.0)
15	Anterior TWI	Negative					3	0 (0.0)	0 (0.0)
16	Anterior TWI	Negative					3	0 (0.0)	0 (0.0)

**Fig. 2:**
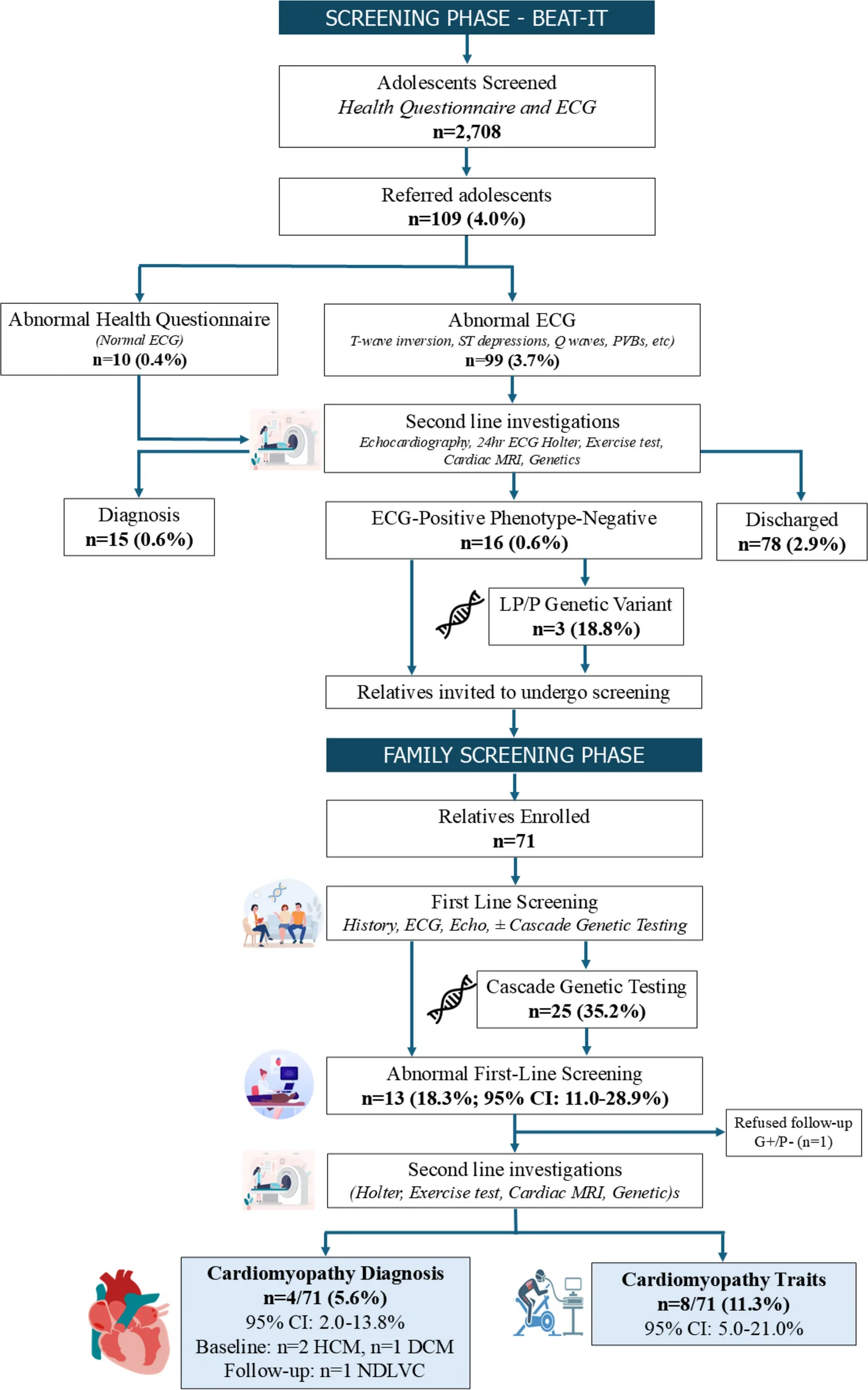
Flow diagram summarizing the BEAT-IT screening pathway for ECG-positive phenotype-negative adolescents and their families

The commonest ECG changes were anterior TWI (*n* = 6, 37.5%) that persisted at 16 years of age, followed by lateral TWI (*n* = 5, 31.3%), isolated inferior TWI (*n* = 3, 18.8%), anterior/inferior TWI (*n* = 1, 6.3%), and ST segment depression (*n* = 1, 6.3%). A genetic variant was identified in 9/16 of these individuals. Three individuals (18.8%) were G + , harboring a LP/P cardiomyopathy variant (Table 1 and Fig. 2). All LP/P variants had a very low prevalence (or never identified) in the local population, including two probands (12.5%) with novel LP variants. None of the probands was given a diagnosis after genotyping. Families (*n* = 3/16, 18.8%) of probands with a LP/P variant underwent a more personalized evaluation.

### Baseline characteristics and clinical screening of family members

Seventy-one individuals from 16 different families gave their consent for clinical screening. Most were female (*n* = 42, 59.2%) with a mean age of 40 ± 16 years at the first evaluation. The vast majority were first-degree relatives (*n* = 48, 67.6%). All relatives were of white ethnicity. A small proportion (*n* = 2, 2.8%) reported cardiac symptoms. Only 6 (8.5%) were adolescents at the first assessment (Table 2).

**Table 2:** Outcome of second-line investigations at baseline assessment (relatives)

Imaging modality	Performed (*n*)	Abnormal (*n*, %)	Abnormality (*n*)
24-h ambulatory ECG monitor	13	3 (23.1)	Triplet: 1 High PVB burden: 2
Cardiopulmonary stress testing	12	4 (30.8)	↓ VO2Max (< 85%): 4 ↓ VO2/WR: 2 Abnormal O2/pulse: 2 ECG changes: 1 Ventricular arrhythmias: 2
Cardiac magnetic resonance	12	7 (58.3)	LV hypertrophy: 4 Dilated LV: 1 High T1 values: 1 Ischemic fibrosis: 1 Non-ischemic fibrosis: 1
Invasive coronary angiography	3	3 (100.0)	Needing revascularization: 1 Medical therapy: 2

The baseline clinical evaluation was normal in most screened relatives (*n* = 58/71, 81.7%). The ECG and echocardiogram were abnormal in 9 (12.7%; 95% CI 6.8–22.4%) and 8 (11.3%; 95% CI 6.8–22.4%) individuals respectively. Seven individuals with an abnormal ECG also had an abnormal echocardiogram (*n* = 7/13, 53.8%). Family members (*n* = 25/71, 35.2%) of ECG-positive phenotype-negative probands who also harbored a LP/P genetic variant were also counseled on cascade testing. Ten (14.1%) family members were G + for the familial LP/P genetic variant.

### Referred family members

Thirteen (18.3%; 95% CI 11.0–28.9%) were referred for a more comprehensive evaluation based on their preliminary clinical evaluation (Fig. 2). Relatives with a LP/P genetic variant (*n* = 9/71, 12.7%) were also referred for second-line investigations, irrespective of the outcome of the initial clinical screen. One of these individuals refused further testing and was excluded from further analysis.

The ECG was the commonest reason for referral (*n* = 9/13, 69.2%), including two symptomatic individuals. The commonest ECG abnormality was TWI (*n* = 3 inferolateral, *n* = 1 anterolateral), followed by ST depression (*n* = 2), PVBs (*n* = 2), and Q waves (*n* = 1). Several individuals had an abnormal echocardiogram (*n* = 8/13, 61.5%). Echocardiography identified left ventricular hypertrophy in 7 relatives. A dilated left ventricle with reduced ejection fraction was observed in another relative. The outcomes of second-line investigations are tabulated in Table 2.

### Baseline diagnostic yield

**Fig. 3:**
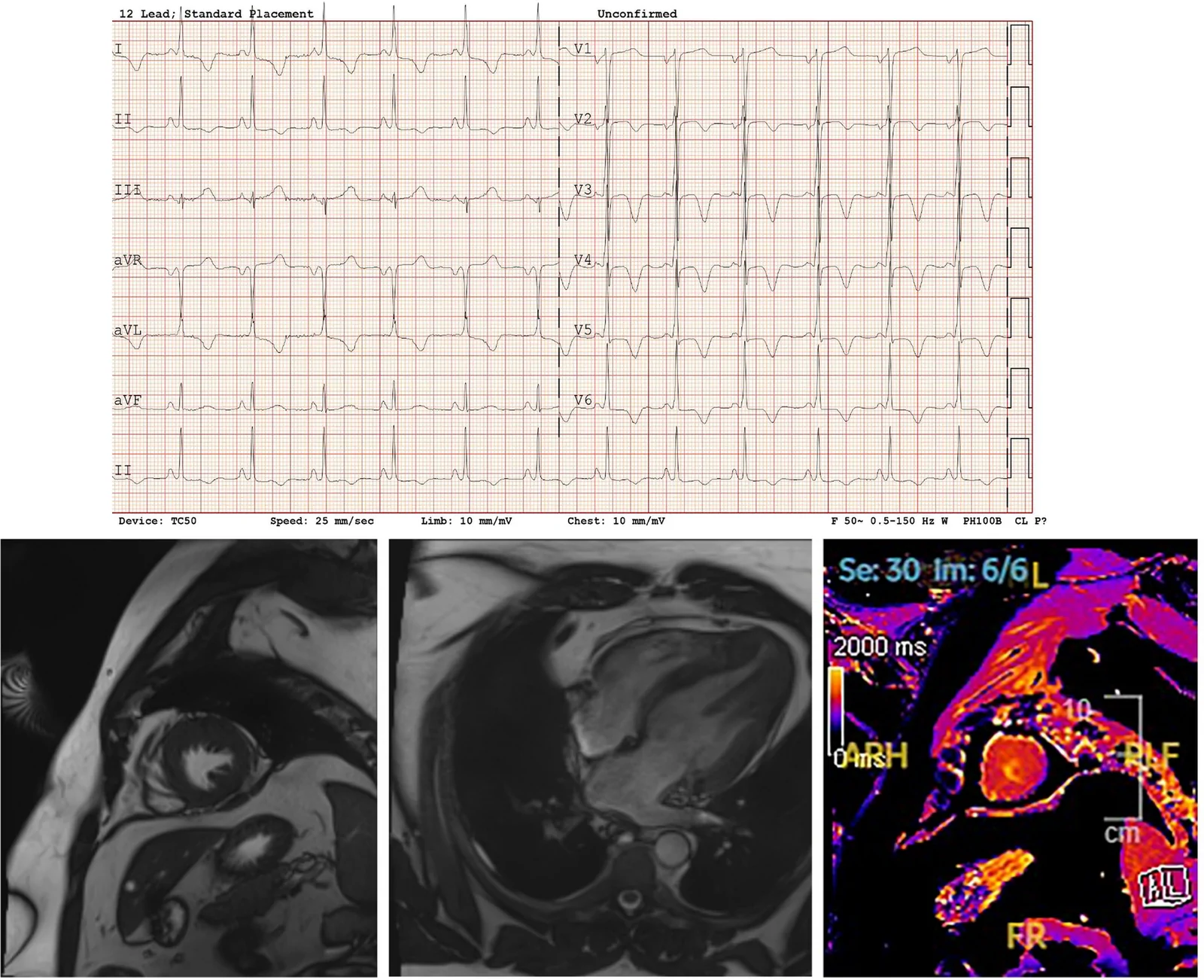
ECG and CMR imaging from a first-degree relative harboring a likely pathogenic MYH7 variant, diagnosed with hypertrophic cardiomyopathy

Following comprehensive testing of all relatives, 3/71 relatives (4.2%; 95% CI 0.9–11.7%), unrelated individuals were given a cardiomyopathy diagnosis (*n* = 2 hypertrophic cardiomyopathy [HCM], *n* = 1 DCM) at the baseline evaluation (Fig. 2). These individuals all had an abnormal resting ECG and echocardiogram (Fig. 3). A LP/P variant was identified in 2 of the 3 individuals.

A further 9 (12.7%; 95% CI 4.9–20.4%) relatives exhibited early cardiomyopathy traits (*n* = 9) (Fig. 2). All these individuals are under active follow-up. One G + /P − relative had a normal ECG and echocardiography, and this individual refused referral for more advanced phenotyping and surveillance.

**Fig. 4:**
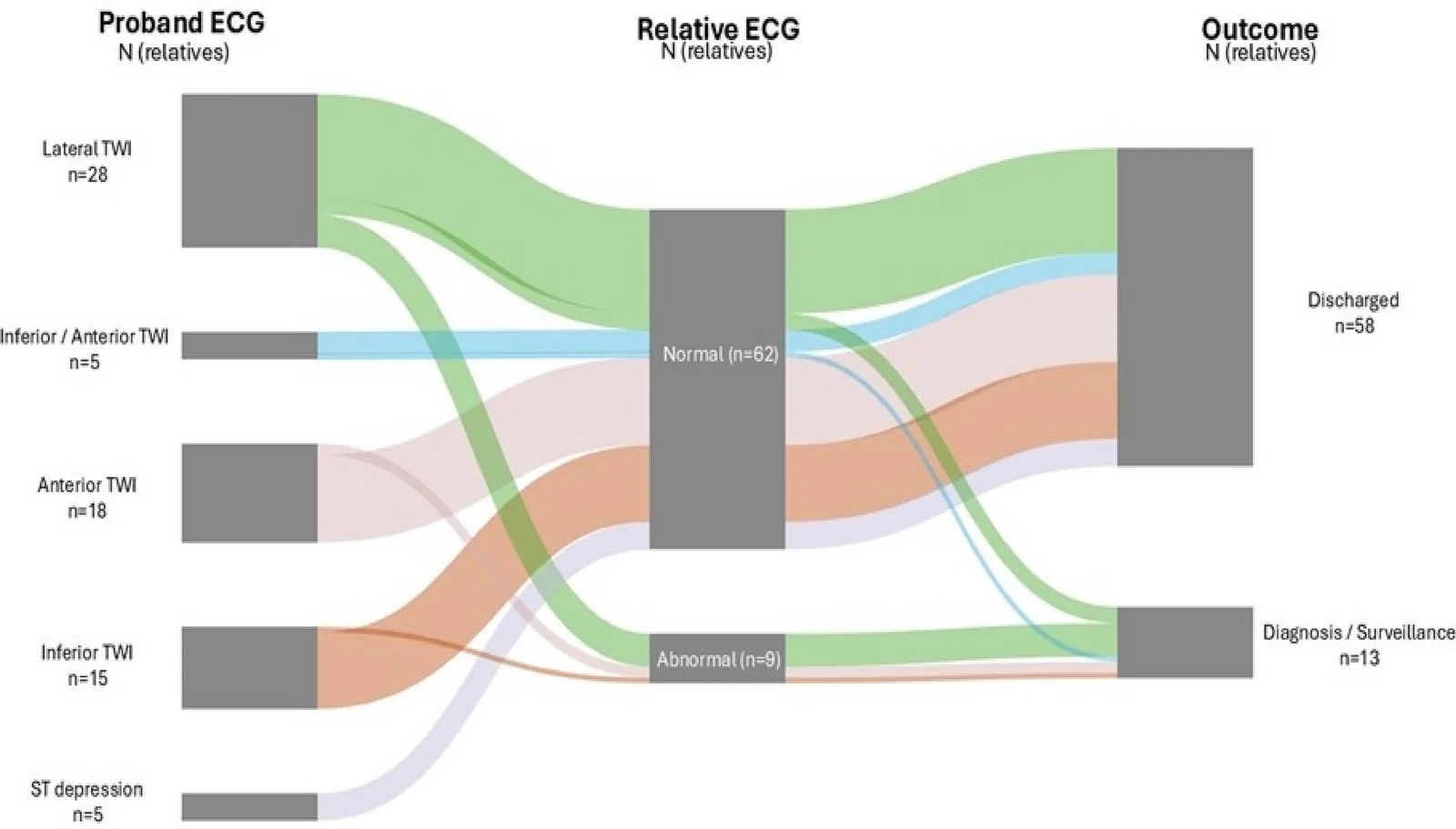
Sankey diagram illustrating the outcome of family screening according to the proband’s ECG pattern and the relatives’ ECG (ECG, electrocardiogram; TWI, T wave inversion)

The presence of lateral TWI and/or LP/P genetic variant in ECG-positive phenotype-negative adolescent probands, a family history of SCD, and relatives with symptoms were more likely to exhibit early cardiomyopathy traits or a diagnosis (Table 3) (Fig. 4).

**Table 3:** Demographic and clinical characteristics in relatives with a diagnosis/under surveillance vs those with a normal evaluation predictors for an abnormal outcome at family screening

Characteristic	Diagnosis/surveillance (*n* = 13) (*n*, %)	Normal evaluation (*n* = 58) (*n*, %)
Female	5 (38.5)	37 (63.8)
Age at 1st evaluation (mean ± SD)	40 ± 16	40 ± 16
≤ 18 years	2 (15.4)	4 (6.9)
White ethnicity	13 (100.0)	58 (100.0)
First-degree relative	9 (69.2)	39 (67.2)
Proband lateral T wave inversion	9 (69.2)	19 (32.8)
Family history of SCD	6 (46.2)	5 (8.6)
Proband LP/P Genetic Variant	8 (61.5)	15 (25.9)
Symptomatic relative	2 (15.4)	0 (0.0)
Lateral T wave inversion (relatives)	4 (30.8)	0 (0.0)

**Fig. 5:**
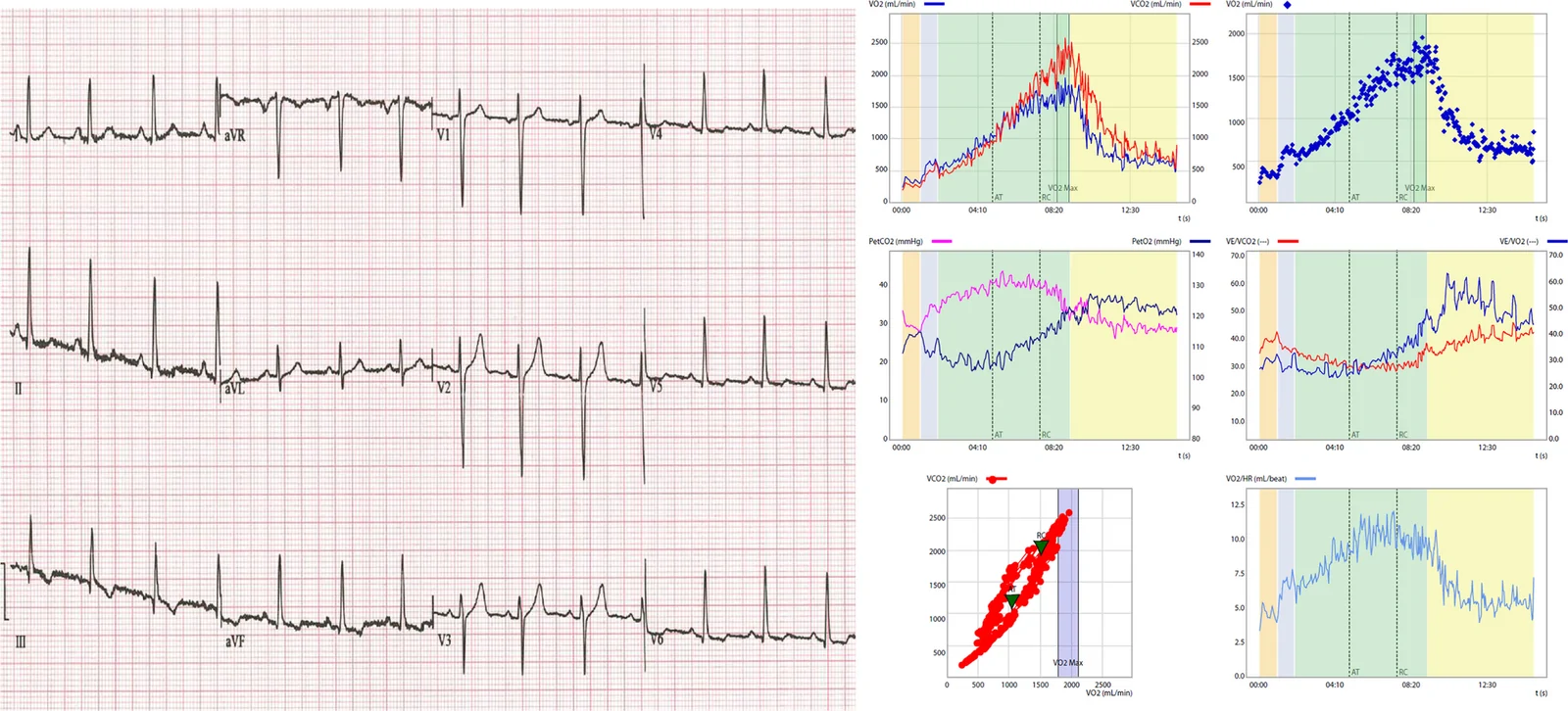
First-degree relative with inferolateral T wave inversion on ECG and early cardiomyopathic traits on her cardiopulmonary stress ECG, in the context of her harboring the familial ACTC1 variant

Relatives requiring surveillance (*n* = 10) often had an abnormal ECG (*n* = 6; lateral TWI *n* = 3, PVBs *n* = 2, ST depression *n* = 1). Most of these individuals (*n* = 9, 12.7%; 95% CI 6.8–22.4%), from six families, also exhibited early cardiomyopathy traits during CPET, ambulatory ECG monitoring, and/or CMR (Table 2) (Fig. 5).

### Screening adolescent relatives

Six (8.%) adolescent relatives were screened in the program. Most were female (*n* = 4/6, 66.7%) and first-degree relatives (*n* = 5/6, 83.3%). Two (33.3%) of these individuals from the same family were G + and exhibited early cardiomyopathy traits (*n* = 1 inferolateral TWI, *n* = 1 evidence of cardiac limitation during CPET). The remaining adolescent relatives had a normal ECG and echocardiogram (gene-negative families).

### Incidental diagnosis

An incidental diagnosis was also identified in another 3 (4.2%) relatives, all unrelated to genetic cardiomyopathy (*n* = 2 hypertensive cardiomyopathy, *n* = 1 ischaemic cardiomyopathy, *n* = 1 LVOT ectopy). The relative with LVOT ectopy had his PVB burden suppressed early on during exercise testing. CMR imaging was also reassuringly normal.

### Management strategy

All three (4.2%) relatives diagnosed with cardiomyopathy required medical therapy at the time of diagnosis (Fig. 2). Two underwent invasive coronary angiography because of symptoms and were both found to have concomitant coronary artery disease. One required revascularization with complete resolution of symptoms. One patient with HCM received an implantable cardioverter defibrillator because of high-risk clinical markers.

### Screening non-immediate family members

Consistent with current recommendations [12, 17], cascade genetic testing was performed in families of patients with a LP/P cardiomyopathy genetic variant. As per the study protocol, the presence of a LP/P variant in first-degree family members (even in the absence of a diagnosis or early cardiomyopathy traits) led to cascade genetic testing in non-immediate (to proband) relatives (*n* = 14, 19.7%). This approach led to a second-degree relative with a new DCM diagnosis (*n* = 1, 1.4%). A further 3 (4.2%) individuals also harbored early cardiomyopathy traits (Fig. 5).

### Follow-up

**Fig. 6:**
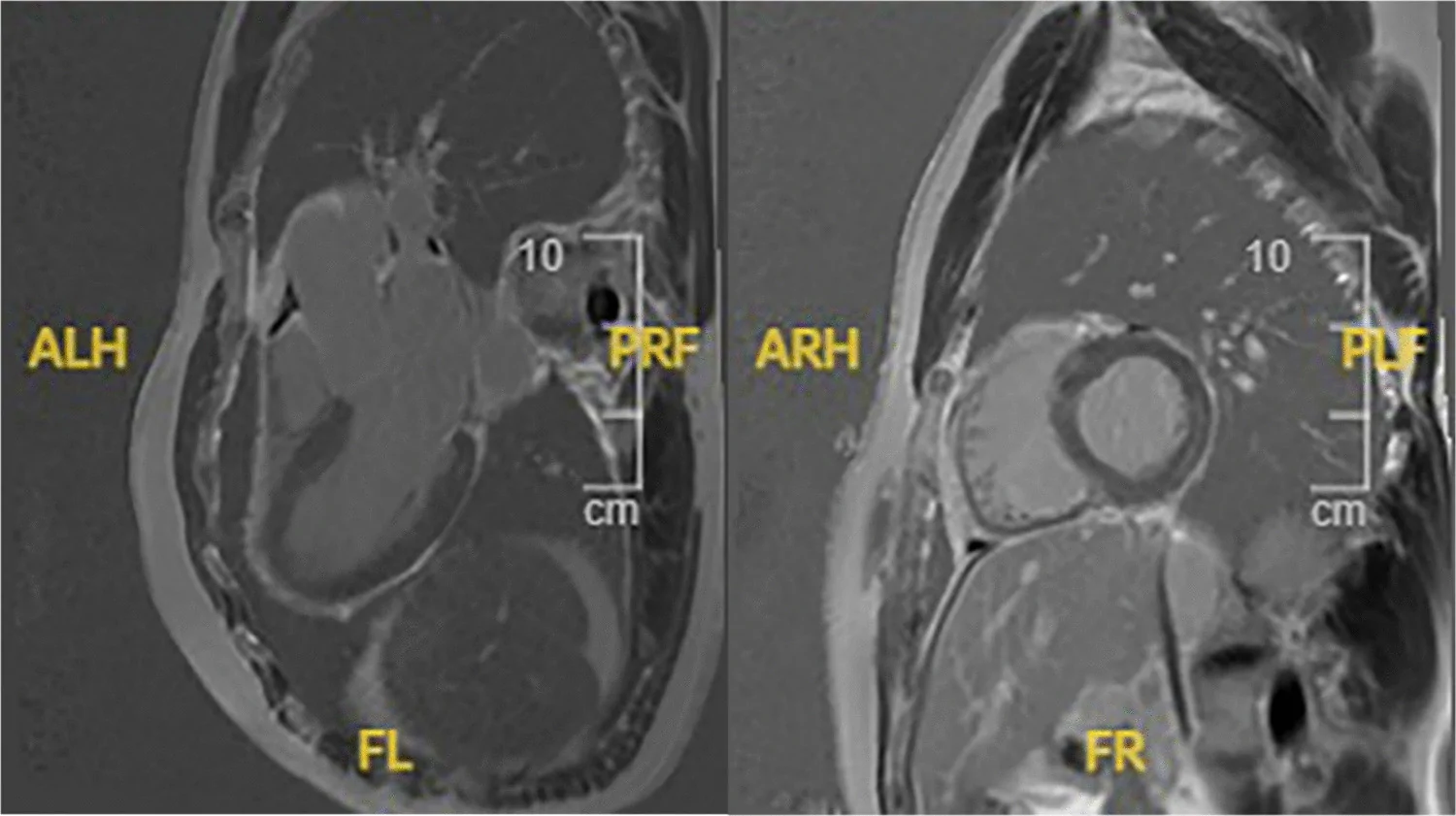
New LV scar on repeating CMR imaging in a first-degree relative with a MYBPC3 pathogenic variant

All relatives with a diagnosis or those having early cardiomyopathy traits underwent active follow-up (59 ± 23 months). One individual with early cardiomyopathy traits at baseline (*n* = 1/9, 11.1%) underwent phenoconversion at 71 months (Figs. 2 and 6). This individual harbored a MYBPC3 P variant, together with cardiomyopathy traits during exercise testing. A repeat CMR identified a new isolated non-ischemic left ventricular mid wall scar in the lateral wall. At the end of the follow-up period, the diagnostic yield for cardiomyopathy diagnosis increased to 5.6% (95% CI 2.0–13.8%). Follow-up CMR in the remaining relatives undergoing surveillance did not identify any new pathology. None of these individuals required any active therapy.

### Gene-positive ECG-positive adolescent probands–how does it all come together?

Three (18.8%) ECG-positive phenotype-negative adolescents probands were identified with a LP/P genetic variant (Table 1). These individuals exhibited inferior, inferolateral, and anterior/inferior TWI. Genotype (LP/P) was not associated with any specific ECG pattern. At the end of the follow-up period, the presence of a LP/P genetic variant in screened relatives was however associated with a higher likelihood of a diagnosis (*n* = 4/10 [40.0%) vs *n* = 0/61 [0.0%)]) or cardiomyopathy traits (*n* = 4/10 [40.0%] vs *n* = 4/61, [6.6%]) (*p* < 0.001). Cascade testing enabled personalized surveillance in 6 relatives (8.5%). These individuals either demonstrated a normal clinical screen (ECG and echocardiography) or were non-immediate relatives who would not have required screening by conventional criteria. Two (2.8%) of these individuals were given a diagnoses, and these would otherwise have been missed.

## Discussion

Electrocardiographic abnormalities may represent one of the earliest phenotypic expression of cardiomyopathy, preceding the morphofunctional changes that typically underpin diagnosis [47, 48]. Current guidelines recommend family screening only when a proband fulfils diagnostic criteria for cardiomyopathy [12, 49]. Cascade genetic testing is traditionally reserved for families in whom a LP/P variant has been identified [12, 30, 49]. Repolarization anomalies suggestive of underlying cardiomyopathy are uncommon when screening adolescents [9, 11, 42, 48, 50], hence why caution is warranted in such cases. Cardiomyopathies often present in the fourth or fifth decade of life [13, 21, 22, 30, 37, 51, 52]. A diagnosis in ECG-positive adolescents is understandably infrequent. Family screening in this context may be reasonable, though its broader applicability remains uncertain. Our observations indicate that clinically relevant anomalies may emerge in selected families, even when adolescents are ECG-positive phenotype-negative on standard screening modalities. These signals are preliminary and may simply reflect cohort enrichment, warranting further evaluation in larger studies.

As a follow-up to the BEAT-IT study [11], we report the clinical impact of family screening in ECG-positive phenotype-negative adolescents. Nearly one in five relatives (18.3%, 95% CI 11.0–28.9%) required second-line investigations. A cardiomyopathy diagnosis was present in 3 individuals (4.2%; 95% CI 0.9–11.7%) at baseline, increasing to 5.6% (95% CI 2.0–13.8%) at the end of the follow-up period. Another 8 individuals (11.3%; 95% CI 4.9–21.0%) had early cardiomyopathy phenotypic traits. An abnormal screening outcome was more prevalent in families where the ECG-positive phenotype-negative adolescent proband had lateral TWI or a LP/P genetic variant, in families with a history of SCD, symptomatic relatives, and in relatives with lateral TWI. Most referred relatives had ECG anomalies, reinforcing the central role of the ECG as an early marker of disease expression within families [51]. Relatives given a diagnosis underwent lifestyle modification, medical therapy, or invasive procedures (diagnostic or preventive therapies), underscoring the clinical relevance of detecting disease in this unconventional clinical context.

Genetic testing in this cohort added further value to the screening process. A fifth of probands harbored rare LP/P variants, not previously identified in the local population. This relatively high yield likely reflects the composition of the cohort, consisting of ECG-positive phenotype-negative adolescents referred for specialist evaluation, rather than an unselected population that is reported in genetic biobank studies [19, 52]. This should not be interpreted as representative of the general adolescent population. Incorporating genetic data in this cohort enabled a more personalized surveillance strategy in 8.5% of relatives. Several relatives with normal ECG and echocardiographic findings demonstrated early cardiomyopathy traits on advanced testing, including morphofunctional and tissue characterization abnormalities (CMR), arrhythmias (holter monitoring/exercise testing), evidence of a cardiac limitation during CPET (Table 2). Two (2.8%) family members were diagnosed with cardiomyopathy (*n* = 1 DCM in non-immediate family member; *n* = 1 NDLVC in a first-degree relative), findings that would have been missed using the conventional screening approach. A diagnosis or early cardiomyopathy traits were also more frequent in G + families (80.0 vs 4.4%, *p* < 0.001), suggesting that a joint clinical-genetic approach may offer additional diagnostic yield in selected contexts. These observations remain exploratory, and should be interpreted cautiously. Our findings are directionally consistent with emerging genotype-first data indicating that carriers of cardiomyopathy-associated variants may exhibit subtle or concealed phenotypes detectable only with advanced imaging or functional assessment [18, 19, 53–55]. Variant carriers have also been reported to have an increased risk of heart failure, cardiomyopathy, and arrythmias [55], reinforcing the concept that G + individuals may benefit from more comprehensive evaluation and closer surveillance. Validation in larger, unselected cohorts will be essential, as genotype-specific or family-specific factors may underlie the differences observed in our cohort.

Although clinically appealing, several barriers may limit implementation. Clinical penetrance among G + individuals is highly heterogenous and often unpredictable. While predictive testing has been shown to be a cost-effective in other settings [56], the financial implications of cascade genetic testing in the absence of established familial cardiomyopathy remains uncertain. The emotional impact of genetic testing in young individuals must also be considered, as a positive result, whether diagnostic or equivocal, may carry biological, psychological, and social consequences [57, 58]. Repeat screening, particularly in G + families, may ultimately increase the diagnostic yield [13, 39], potentially strengthening the long-term economic justification for this approach. Nonetheless, our data should be interpreted as hypothesis-generating rather than definitive, given the modest sample size and small number of events.

### Limitations

This study has some limitations. The proband sample size is modest, reflecting the low prevalence of ECG-positive phenotype-negative adolescents in the screening program. The population is predominantly of white ethnicity, limiting generalizability, particularly given the higher prevalence of TWI in Afrocarribean individuals [42, 59–61]. The absence of a comparator group also makes it difficult to quantify the incremental diagnostic yield above background detection rates.

The genetic architecture of the Maltese population, including potential founder effects, may also influence variant distribution and partly explain the relatively high genetic yield, with the impact of founder mutations in island communities already described previously by local and international academic groups [62–64]. Despite the clinically actionable genetic variants identified in this study carrying a low prevalence in the local population, founder effects may possibly explain the comparatively high LP/P yield in phenotype-negative probands.

Penetrance of LP/P genetic variants is incomplete and age-dependent. We did not perform a cost-effectiveness analysis to justify this novel screening approach. Although our findings support the feasibility and potential direction of genotype-guided surveillance in selected families, these findings should be considered as hypothesis-generating. The role of routine genetic testing in all ECG-positive phenotype-negative adolescents require confirmation in larger cohorts.

The cohort of relatives was drawn from 16 families, meaning that certain clinical and genetic characteristics were shared across individuals. Within-family clustering was not modeled, as the study was descriptive rather than inferential. This may reduce statistical independence and attenuate *p*-values; however, it does not alter the descriptive patterns observed nor the exploratory intent of the study.

Although no MACE occurred during follow-up, this study was neither powered nor designed to asses prognostic benefit. Our results merely highlight the potential benefit of this approach to identify more family members with early phenotypic cardiomyopathy traits or a diagnosis. Longer surveillance may in turn reveal further phenotypic evolution. Extended follow-up will also shed light on the long-term implications of harboring a LP/P cardiomyopathy variant, in the absence of overt cardiomyopathy.

Despite these limitations, our findings highlight the potential value of a combined clinical and genetic approach in families of adolescents with ECG-positive phenotype-negative adolescents. Early identification of at-risk relatives may enable timely surveillance, risk stratification, and intervention. Larger studies are needed to refine and validate the role of genetic testing in this setting and to determine which subgroups derive the greatest benefit from extended family evaluation.

## Conclusion

This exploratory, proof of concept study examined the yield of clinical and genetic screening in families of ECG-positive, phenotype-negative adolescents that may be harboring pre-clinical cardiomyopathy. Repolarization ECG changes suggestive of cardiomyopathy are rare in adolescents [9, 11, 13, 48, 50], hence why these findings warrant referral for more advanced testing. Given the age-dependent penetrance of inherited cardiomyopathies, adolescents rarely meet diagnostic criteria, making long-term surveillance a reasonable consideration. In this context, family screening may be justified. The presence of a family history of SCD, lateral TWI or a LP/P cardiomyopathy variant in ECG-positive phenotype-negative adolescents, and symptomatic relatives in this cohort was associated with a higher yield when screening relatives. In this enriched cohort, family screening identified a small proportion of individuals with a new cardiomyopathy diagnosis (5.6%) and early cardiomyopathic traits (11.3%). Our findings support that a combined clinical-genetic approach may offer additional insight and could support more individualized screening strategies in selected families. These observations remain preliminary and hypothesis-generating. These should be validated in larger, unselected cohorts, as genotype-specific or family-specific factors may have contributed to these findings.

## Data Availability

No datasets were generated or analysed during the current study.

## Declarations

All authors have nothing to declare.

## References

[1] Finocchiaro G, Radaelli D, D’Errico S et al (2023) Sudden cardiac death among adolescents in the united Kingdom. J Am Coll Cardiol 81(11):1007–1017. 10.1016/j.jacc.2023.01.041 36922085

[2] Corrado D, Schmied C, Basso C et al (2011) Risk of sports: do we need a pre-participation screening for competitive and leisure athletes? Eur Heart J 32(8):934–944. 10.1093/eurheartj/ehq482 21278396

[3] Drezner JA, Connor FGO, Harmon KG et al (2016) Position statement on cardiovascular preparticipation screening in athletes: current evidence, knowledge gaps, recommendations, and future directions. Clin J Sports Med 26(5):347–361 10.1097/JSM.0000000000000382 27598018

[4] Corrado D, Basso C, Schiavon M, Pelliccia A, Thiene G (2008) Pre-participation screening of young competitive athletes for prevention of sudden cardiac death. J Am Coll Cardiol 52(24):1981–1989. 10.1016/j.jacc.2008.06.053 19055989

[5] Corrado D, Basso C, Rizzoli G, Schiavon M, Thiene G (2003) Does sports activity enhance the risk of sudden death in adolescents and young adults? J Am Coll Cardiol 42(11):1959–1963. 10.1016/j.jacc.2003.03.002 14662259

[6] Corrado D, Basso C, Pavei A, Michieli P, Schiavon MTG (2006) Trends in sudden cardiovascular death in young competitive athletes. JAMA 296(13):1593–1601. 10.1001/jama.296.13.1593 17018804

[7] Marijon E, Tafflet M, Celermajer DS et al (2011) Sports-related sudden death in the general population. Circulation 124(6):672–681. 10.1161/CIRCULATIONAHA.110.008979 21788587

[8] Drezner JA, Toresdahl BG, Rao AL, Huszti E, Harmon KG (2013) Outcomes from sudden cardiac arrest in US high schools: a 2-year prospective study from the National Registry for AED Use in Sports. Br J Sports Med 47(18):1179–1183. 10.1136/bjsports-2013-092786 24124037

[9] Malhotra A, Dhutia H, Finocchiaro G et al (2018) Outcomes of cardiac screening in adolescent soccer players. N Engl J Med 379(6):524–534. 10.1056/NEJMoa1714719 30089062

[10] Dhutia H, Malhotra A, Finocchiaro G et al (2021) Diagnostic yield and financial implications of a nationwide electrocardiographic screening programme to detect cardiac disease in the young. EP Europace. 23(8):1295–1301. 10.1093/europace/euab021 PMC8350863 33570096

[11] Abela M, Yamagata K, Bonello J et al (2024) BEAT-IT: a de-novo cardiac screening programme in Maltese adolescents. Hellenic J Cardiol 79:49–57. 10.1016/j.hjc.2023.09.012 37743018

[12] Arbelo E, Protonotarios A, Gimeno JR et al (2023) 2023 ESC guidelines for the management of cardiomyopathies. Eur Heart J 44(37):3503–3626. 10.1093/eurheartj/ehad194 37622657

[13] Sarto P, Zorzi A, Merlo L et al (2021) Serial versus single cardiovascular screening of adolescent athletes. Circulation 143(17):1729–1731. 10.1161/CIRCULATIONAHA.120.053168 PMC8061335 33900830

[14] Zeppenfeld K, Tfelt-Hansen J, de Riva M et al (2022) 2022 ESC guidelines for the management of patients with ventricular arrhythmias and the prevention of sudden cardiac death. Eur Heart J 43(40):3997–4126. 10.1093/eurheartj/ehac262 36017572

[15] Mcgorrian C, Constant O, Harper N et al (2013) Family-based cardiac screening in relatives of victims of sudden arrhythmic death syndrome. Europace 15:1050–1058. 10.1093/europace/eus408 23382499

[16] Te Riele ASJM, James CA, Groeneweg JA et al (2016) Approach to family screening in arrhythmogenic right ventricular dysplasia/cardiomyopathy. Eur Heart J 37(9):755–763. 10.1093/eurheartj/ehv387 26314686

[17] Lafreniere-Roula M, Bolkier Y, Zahavich L et al (2019) Family screening for hypertrophic cardiomyopathy: is it time to change practice guidelines? Eur Heart J 0:1–10. 10.1093/eurheartj/ehz396 PMC6885133 31170284

[18] Asatryan B, Murray B, Tadros R et al (2024) Promise and peril of a genotype-first approach to mendelian cardiovascular disease. J Am Heart Assoc 13(21):e033557. 10.1161/JAHA.123.033557 PMC11935662 39424414

[19] Shah RA, Asatryan B, SharafDabbagh G et al (2022) Frequency, penetrance, and variable expressivity of dilated cardiomyopathy-associated putative pathogenic gene variants in UK Biobank participants. Circulation 146(2):110–124. 10.1161/CIRCULATIONAHA.121.058143 PMC9375305 35708014

[20] Caselli S, Pelliccia A (2019) The electrocardiogram and the phenotypic expression of hypertrophic cardiomyopathy. Eur Heart J 40(12):982–985. 10.1093/eurheartj/ehy403 30010740

[21] Jensen MK, Havndrup O, Christiansen M et al (2013) Penetrance of hypertrophic cardiomyopathy in children and adolescents: a 12-year follow-up study of clinical screening and predictive genetic testing. Circulation 127(1):48–54. 10.1161/CIRCULATIONAHA.111.090514 23197161

[22] Lorenzini M, Norrish G, Field E et al (2020) Penetrance of Hypertrophic Cardiomyopathy in Sarcomere Protein Mutation Carriers. J Am Coll Cardiol 76(5):550–559. 10.1016/j.jacc.2020.06.011 PMC7397507 32731933

[23] Lakdawala NK, Thune JJ, Maron BJ et al (2011) Electrocardiographic features of sarcomere mutations carriers with versus without clinically overt hypertrophic cardiomyopathy. Am J Cardiol 108(11):1606–1613. 10.1016/j.amjcard.2011.07.019 PMC3215918 21943931

[24] Lukas Laws J, Lancaster MC, Ben Shoemaker M et al (2022) Arrhythmias as presentation of genetic cardiomyopathy. Circ Res 130(11):1698–1722. 10.1161/CIRCRESAHA.122.319835 PMC9205615 35617362

[25] Stroeks SLVM, Wang P, Merlo M et al (2025) Impact of genotype–phenotype associations on prognosis in dilated cardiomyopathy. Eur J Heart Fail. 10.1002/ejhf.70040 PMC12803617 40938777

[26] Limongelli G, Nunziato M, D’Argenio V et al (2021) Yield and clinical significance of genetic screening in elite and amateur athletes. Eur J Prev Cardiol 28(10):1081–1090. 10.1177/2047487320934265 34425588

[27] Johnson R, Fletcher RA, Peters S et al (2025) Titin-related familial dilated cardiomyopathy: factors associated with disease onset. Eur Heart J 46(48):5240–5257. 10.1093/eurheartj/ehaf380 PMC12718689 40796136

[28] Hasselberg NE, Haland TF, Saberniak J et al (2018) Lamin A/C cardiomyopathy: young onset, high penetrance, and frequent need for heart transplantation. Eur Heart J 39(10):853–860. 10.1093/eurheartj/ehx596 PMC5939624 29095976

[29] Shah RA, Asatryan B, Dabbagh GS et al (2022) Frequency, penetrance, and variable expressivity of dilated cardiomyopathy-associated putative pathogenic gene variants in UK Biobank participants. Circulation. 10.1161/CIRCULATIONAHA.121.058143 PMC9375305 35708014

[30] Verdonschot JAJ, Kaski JP, Asselbergs FW et al (2025) Clinical care of family members of patients with dilated cardiomyopathy. Eur Heart J. 10.1093/eurheartj/ehaf571 PMC12614981 40902100

[31] Muller SA, Gasperetti A, Bosman LP et al (2023) Individualized family screening for arrhythmogenic right ventricular cardiomyopathy. J Am Coll Cardiol 82(3):214–225. 10.1016/j.jacc.2023.05.005 37210036

[32] Thierry IP, Muller SA, Baas AF et al (2025) Yield of family screening for dilated cardiomyopathy: 10-year experience at a multidisciplinary cardiogenetic outpatient clinic. Neth Hear J 33(2):46–54. 10.1007/s12471-024-01924-1 PMC11757811 39833651

[33] Puchnerova V, Jensovsky M, Zoubkova V et al (2026) The Long-Term Clinical Progression of Genotype-Positive/Phenotype-Negative Patients With Hypertrophic Cardiomyopathy. Am J Cardiol 267:1–6. 10.1016/j.amjcard.2026.02.042 41759561

[34] van Velzen HG, Schinkel AFL, Baart SJ et al (2018) Outcomes of contemporary family screening in hypertrophic cardiomyopathy. Circ Genom Precis Med. 10.1161/CIRCGEN.117.001896 29661763

[35] Silajdzija E, Vissing CR, Christensen EB et al (2024) Family Screening in Hypertrophic Cardiomyopathy. J Am Coll Cardiol 84(19):1854–1865. 10.1016/j.jacc.2024.08.011 39365224

[36] Gray B, Ingles J, Semsarian C (2011) Natural history of genotype positive–phenotype negative patients with hypertrophic cardiomyopathy. Int J Cardiol 152(2):258–259. 10.1016/j.ijcard.2011.07.095 21862152

[37] Nielsen SK, Hansen FG, Rasmussen TB et al (2023) Major cardiac events in patients and relatives with hereditary hypertrophic cardiomyopathy. JACC Adv 2(8):100604. 10.1016/j.jacadv.2023.100604 PMC11198269 38938358

[38] Abdulrahim JW, Kwee LC, Alenezi F et al (2020) Identification of undetected monogenic cardiovascular disorders. J Am Coll Cardiol 76(7):797–808. 10.1016/j.jacc.2020.06.037 PMC7428466 32792077

[39] Cannie D, Syrris P, Protonotarios A et al (2023) Disease penetrance in genotype-positive relatives from families with dilated cardiomyopathy. Eur Heart J. 10.1093/eurheartj/ehad655.1864 40848704

[40] Orchard J, Harmon KG, D’Ascenzi F, Meyer T, Pieles GE (2024) What is the most appropriate age for the first cardiac screening of athletes?. J Sci Med Sport. Published online June 2024. 10.1016/j.jsams.2024.05.017 38890019

[41] Ahimaz P, Sabatello M, Qian M et al (2021) Impact of genetic testing for cardiomyopathy on emotional well-being and family dynamics: a study of parents and adolescents. Circ Genom Precis Med 14(4):E003189. 10.1161/CIRCGEN.120.003189 PMC8373687 34255550

[42] Drezner JA, Sharma S, Baggish A et al (2017) International criteria for electrocardiographic interpretation in athletes : consensus statement. Br J Sports Med 51:704–731. 10.1136/bjsports-2016-097331 28258178

[43] Ackerman MJ, Priori SG, Willems S et al (2011) HRS/EHRA Expert Consensus Statement on the State of Genetic Testing for the Channelopathies and Cardiomyopathies. Heart Rhythm 8(8):1308–1339. 10.1016/j.hrthm.2011.05.020 21787999

[44] Etheridge SP, Saarel EV, Martinez MW (2018) Exercise participation and shared decision-making in patients with inherited channelopathies and cardiomyopathies. Heart Rhythm 15(6):915–920. 10.1016/j.hrthm.2017.12.020 29248563

[45] Towbin JA, Mckenna WJ, Vice-chair D et al (2019) 2019 HRS expert consensus statement on evaluation, risk stratification, and management of arrhythmogenic cardiomyopathy. Heart Rhythm 16(11):e301-372. 10.1016/j.hrthm.2019.05.007 31078652

[46] Richards S, Aziz N, Bale S et al (2015) Standards and guidelines for the interpretation of sequence variants: a joint consensus recommendation of the American College of Medical Genetics and Genomics and the Association for Molecular Pathology. Genet Med 17(5):405–424. 10.1038/gim.2015.30 PMC4544753 25741868

[47] Aimo A, Milandri A, Barison A et al (2024) Electrocardiographic abnormalities in patients with cardiomyopathies. Heart Fail Rev 29(1):151–164. 10.1007/s10741-023-10358-7 PMC10904564 37848591

[48] Pieles GE, Cavarretta E, Orchard JJ et al (2026) Cardiac evaluation of paediatric athletes. Eur Heart J. 10.1093/eurheartj/ehag188 PMC13225883 41967042

[49] Elliott P, Schunkert H, Bondue A et al (2025) Integration of genetic testing into diagnostic pathways for cardiomyopathies: a clinical consensus statement by the ESC Council on Cardiovascular Genomics. Eur Heart J 46(4):344–353. 10.1093/eurheartj/ehae747 39673718

[50] MacLachlan H, Bhatia R, Raju H et al (2026) Cardiac screening for conditions associated with sudden cardiac death: yield, interventions, and SCA/SCD Incidence in 104,369 young individuals. J Am Coll Cardiol 87(7):756–768. 10.1016/j.jacc.2025.11.049 41738673

[51] Norrish G, Jager J, Field E et al (2019) Yield of clinical screening for hypertrophic cardiomyopathy in child first-degree relatives. Circulation 140(3):184–192. 10.1161/CIRCULATIONAHA.118.038846 PMC6636798 31006259

[52] Josephs KS, Seaby EG, May P et al (2024) Cardiomyopathies in 100,000 genomes project: interval evaluation improves diagnostic yield and informs strategies for ongoing gene discovery. Genome Med 16(1):125. 10.1186/s13073-024-01390-9 PMC11520845 39472908

[53] Claessen G, De Bosscher R, Janssens K et al (2024) Reduced ejection fraction in elite endurance athletes: clinical and genetic overlap with dilated cardiomyopathy. Circulation 149(18):1405–1415. 10.1161/CIRCULATIONAHA.122.063777 PMC11062611 38109351

[54] Sheikh N, Papadakis M, Wilson M, Malhotra A, Sharma S (2018) Diagnostic yield of genetic testing in young athletes with t-wave inversion. Circulation 138(12):1184–1194. 10.1161/CIRCULATIONAHA.118.034208 PMC6147090 29764897

[55] Shetty NS, Pampana A, Gaonkar M et al (2025) Association of Pathogenic/Likely Pathogenic Genetic Variants for Cardiomyopathies With Clinical Outcomes: A Multiancestry Analysis in the All of Us Research Program. Circ Genom Precis Med 18(3):e005113. 10.1161/CIRCGEN.124.005113 40433684

[56] Catchpool M, Ramchand J, Martyn M et al (2019) A cost-effectiveness model of genetic testing and periodical clinical screening for the evaluation of families with dilated cardiomyopathy. Genet Med 21(12):2815–2822. 10.1038/s41436-019-0582-2 PMC6892743 31222143

[57] Collins RE, Lopez LM, Marteau TM (2011) Emotional impact of screening: a systematic review and meta-analysis. BMC Public Health 11(1):603. 10.1186/1471-2458-11-603 PMC3223929 21798046

[58] Ahimaz P, Sabatello M, Qian M et al (2021) Impact of genetic testing for cardiomyopathy on emotional well-being and family dynamics: a study of parents and adolescents. Circ Genom Precis Med. 10.1161/CIRCGEN.120.003189 PMC8373687 34255550

[59] Mcclean G, Riding NR, Pieles G et al (2019) Prevalence and significance of T-wave inversion in Arab and Black paediatric athletes; should anterior T-wave inversion interpretation be governed by biological or chronological age? Eur J Prev Cardiol 26(6):641–652. 10.1177/2047487318811956 30426769

[60] Calore C, Zorzi A, Sheikh N et al (2016) Electrocardiographic anterior T-wave inversion in athletes of different ethnicities: differential diagnosis between athlete’s heart and cardiomyopathy. Eur Heart J 37(32):2515–2527. 10.1093/eurheartj/ehv591 26578198

[61] Wilson MG, Chatard JC, Carre F et al (2012) Prevalence of electrocardiographic abnorm alities in West-Asian and African male athletes. Br J Sports Med 46(5):341–347. 10.1136/bjsm.2010.082743 PMC3329226 21596717

[62] Claes GRF, Van Tienen FHJ, Lindsey P et al (2016) Hypertrophic remodelling in cardiac regulatory myosin light chain (MYL2) founder mutation carriers. Eur Heart J 37(23):1815–1822. 10.1093/eurheartj/ehv522 26497160

[63] Macgregor S, Bellis C, Lea RA et al (2010) Legacy of mutiny on the Bounty: founder effect and admixture on Norfolk Island. Eur J Hum Genet 18:67–72. 10.1038/ejhg.2009.111 PMC2987173 19584896

[64] Scerri CA, Abela W, Galdies R, Pizzuto M, Grech JL, Felice AE (1993) The beta + IVS, I-NT no. 6 (T --> C) thalassaemia in heterozygotes with an associated Hb Valletta or Hb S heterozygosity in homozygotes from Malta. Br J Haematol. 83(4):669-671. 10.1111/j.1365-2141.1993.tb04710.x 8518184

